# Kimura’s disease: a rare cause of facial mass in a caucasian male patient

**DOI:** 10.1259/bjrcr.20200099

**Published:** 2020-08-28

**Authors:** Célia Peixoto Sousa, Elsa Fonseca, Bárbara Viamonte, João Calheiros Lobo, António Madureira

**Affiliations:** 1 Centro Hospitalar de São João, Alameda Prof. Hernâni Monteiro, Porto, Portugal

## Abstract

Kimura’s disease is an uncommon inflammatory disorder of unclear aetiology, mainly affecting young Asian descent males among their second and fourth decades of life. The disease typically emerges as a long-standing and painless subcutaneous mass lesion in the head and neck region, frequently associated with swelling of major salivary glands, particularly the parotid gland, and regional lymphadenopathy. Peripheral eosinophilia and high serum immunoglobulin E are also characteristic findings. We report a case and describe the imaging and pathological features of the disease in a 19-year-old Caucasian male, with review of the literature.

## Case presentation

A 19-year-old Caucasian male patient was referred to our department with complaints of an increasing facial swelling on the left side for 1.5 years, causing aesthetic deformity. The skin overlying the lesion was normal, and the patient did not report pruritus or abnormal bleeding. The lesion was also non-tender and firm at physical examination. The facial motricity and sensation were preserved. The patient was also previously healthy, with no history of drug/medication use, or documented allergies. He did not report other significant complaints.

## Investigations

**Figure 1.**
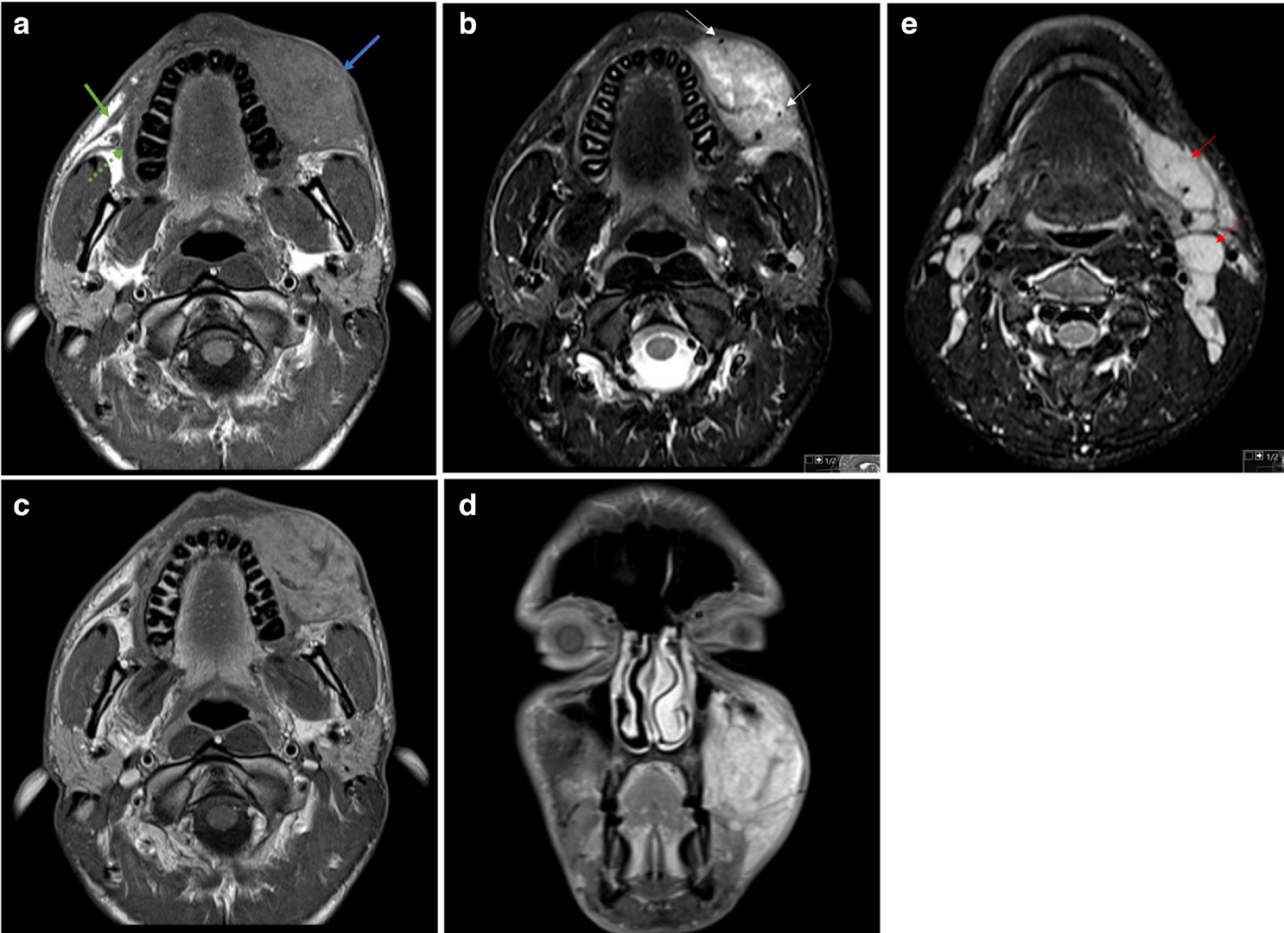
MRI shows a mostly ill-defined mass in the left buccal space (blue arrow), infiltrating the buccinator muscle and adjacent superficial muscles of facial expression. The normal right buccinator (dashed arrow) and zygomaticus muscles (solid green arrow) are depicted for comparison purposes. The lesion appears hyperintense on T1W (A) and STIR (B) images, and shows heterogeneous enhancement on post-contrast T1W images (C and D). The presence of flow-voids is also depicted (arrows in B). Multiple enlarged homogeneous cervical lymph nodes are also present, most prominent on the left side (red arrows in E).

**Figure 2.**
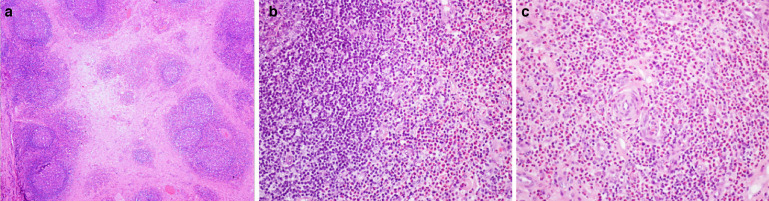
(A) Hyperplastic lymphoid follicles with reactive germinal centres in a fibrotic background (original magnification x40). (B) Dense infiltrate of eosinophils surrounding a lymphoid follicle and (C) in the fibrotic tissue with proliferated vessels (original magnification x200).

A MRI was performed showing a mostly ill-defined mass in the left buccal space, infiltrating adjacent muscles of facial expression, such as the buccinator, levator anguli oris and zygomaticus. The lesion appeared hyperintense on T1W, T2W and STIR images and showed enhancement on post-contrast T1W images (Figure 1). The presence of flow-voids was also depicted. The major salivary glands were unremarkable. Also, multiple enlarged homogeneous cervical lymph nodes were seen at level I, II, and III on the left side and at level II on the right side. A routine blood test showed eosinophilia (26,5%). The surgical biopsy revealed hyperplastic lymphoid follicles with reactive germinal centres in a fibrotic background, a dense mixed inflammatory cellular infiltrate composed mainly of eosinophils and lymphocytes, and a richly vascular stroma (Figure 2). Additional investigations revealed an elevated serum immunoglobulin E (IgE, 2800 UI/mL; N 100 UI/mL) and IgG4 (267 mg/dl; N 140 mg/dl). The laboratory results also showed normal liver and renal function.

## Differential diagnosis

- Kimura’s disease
- Malignancy: lymphoma, soft tissue sarcoma
- Benign tumours: peripheral nerve sheath tumour, desmoid tumour, haemangiopericytoma, angioleiomyoma
- IgG4-related disease

## Treatment

The lesion in the left side of the face was surgically resected. No cervical lymphadenectomy or complimentary post-operative treatment was performed.

## Outcome and follow-up

The pathological examination of the resected lesion was similar to the previous study, confirming the diagnosis of Kimura’s disease. Unfortunately, local relapse was noticed just 2 months after the surgery when the patient started to feel a small lump in the face. Without additional treatments, the lesion registered a progressive growth over 2 years. Recently, the patient was appointed to a specialized consultation in autoimmune diseases.

## Discussion

Kimura’s disease is an uncommon inflammatory disorder of unclear aetiology, with an indolent and chronic course. It was first reported in Chinese literature in 1937 as “eosinophilic hyperplastic lymphogranuloma” by Kimm and Szeto,^1^ and subsequently by Kimura,^2^ who described the histopathological features of the disease. The vast majority of cases are reported in Asia (China, Japan, and Southeast Asia), mainly affecting young Asian descent males during their second and fourth decades of life. However, some sporadic and rare cases have also been described in individuals of Western descent.^3,4^ The disease typically emerges as a long-standing and painless subcutaneous mass lesion in the head and neck region, frequently associated with swelling of major salivary glands, particularly the parotid gland, and regional lymphadenopathy. At presentation, the mass lesions are commonly solitary but sometimes multiple and large, with dimensions greater than 3 cm.^5,6^ Although isolated cervical lymphadenopathy may be the only initial presentation of the disease.^4^ One distinct and intriguing presentation in non-Asian patients is that the salivary glands are infrequently affected,^7^ as observed in our case. Possible locations of disease involvement include the face, neck, periauricular, eyelid, lacrimal gland, pharynx, palate, oral cavity, and, more rarely, the axilla, groin, popliteal region, upper limb and abdomen.^3,4,6,7^ The skin overlying the lesions is generally unremarkable. However, melanin pigmentation and pruritus can be observed in sporadic cases.^6^

The main disease presentation is peripheral eosinophilia (>5 %) and elevated serum IgE, which is almost always present and crucial for auxiliary diagnoses.^4–6^ Although the pathogenesis remains unclear, Kimura’s disease is nowadays considered an allergic reaction and might be triggered by a systemic immunological response due to viral or parasitic infection. A dysregulated CD4(+) T-helper two immune response produces interleukins (IL) IL-4, IL-5 and IL-13, which in turn contribute to marked eosinophilia and high-serum IgE levels.^8^ Additionally, proteinuria may occur in 12–16% of patients with Kimura’s disease, which may progress to nephrotic syndrome and sometimes precede the skin lesions. The renal impairment may be attributed to immune complex-mediated damage or a dysregulated T-cell response, supporting the above hypothesis.^6–8^

Histopathology of the subcutaneous lesions, salivary glands and lymph nodes reveals a prominent and mixed inflammatory cellular infiltrate composed mainly of eosinophils and lymphocytes. The inflammatory infiltrate contains numerous lymphoid follicles with prominent germination centres interspersed with variable degrees of fibrosis. Eosinophilic micro-abscesses are sometimes depicted. The lesions are also formed by a richly vascular stroma, with small arteries, numerous capillaries and proliferation of post-capillary venules. No atypical cells, multinucleated giant cells, necrosis or granulomas are present. Fine-needle aspiration and core-needle biopsy have low diagnostic accuracy, so a biopsy of the surgical specimen is mandatory to confirm the diagnosis.^5,9^

Imaging findings are non-specific and resemble other benign and malignant disorders, but they add value in differential diagnoses and the relationship of the lesions with adjacent structures. MRI is the modality of choice due to the better soft-tissue resolution. The lesions (subcutaneous and salivary gland lesions) appear well-defined or ill-defined/infiltrative, iso- or hyperintense compared to skeletal muscle on T1W images, hyperintense on T2W and STIR images, and show homogeneous or heterogeneous enhancement on post-contrast images, due to variable degrees of fibrosis. The presence of flow voids in the mass lesions reflecting vascular proliferation has also been described. The involved lymph nodes appear more homogeneous without necrosis.^10–12^ The lesions show increased diffusivity, but lower than the lymphadenopathies, with mean ADC values of 1.21 ± 0.33 × 10^-3 11^. Some studies report higher ADC values of the parotid lesions compared to the contralateral normal gland, which can help in the diagnosis of Kimura’s disease rather than malignancy.^10,13^ On CT, the lesions appear hypodense and show homogeneous or heterogeneous enhancement on post-contrast images. Ultrasound examination often shows hypoechoic and enlarged salivary glands with heterogeneous echotexture, and hypoechoic masses of unclear borders, irregular shape, and with a rich blood flow signal. The involved lymph nodes appear enlarged, with preserved central fatty hila and increased hilar vascularity.^3,10,12^

A detailed clinical history along with radiological and pathological correlation is crucial for the correct diagnosis, because none of the features mentioned above is pathognomonic of Kimura’s disease. As a consequence, the differential diagnosis is extensive with a range of reactive and neoplastic processes. Imaging findings may be similar to malignant disorders, such as lymphoma, soft tissue sarcoma and salivary gland malignant tumour, or even benign tumours of the head and neck. Other differential diagnoses on imaging are that of lymph node enlargement and include reactive nodes, mononucleosis, nodal metastases, Castleman’s disease, drug reactions, parasitic infections and IgG4-related disease (IgG4-RD). Peripheral blood eosinophilia may suggest an allergic or parasitic aetiology.^4,7^ The condition most commonly misdiagnosed as Kimura’s disease is angiolymphoid hyperplasia with eosinophilia (ALHE). However, it has distinctive clinical and pathological features. ALHE usually presents as small erythematous dermal papulonodules that bleed easily and can itch. Additionally, it is rarely associated with lymphadenopathy, serum eosinophilia and elevated serum IgE levels.^6,9^ Patients with Kimura’s disease may also exhibit high serum levels of IgG4, as in the case presented. Abundant IgG4-positive plasma cells in the affected tissues are also frequently encountered. This finding can be explained as an epiphenomenon due to the dysregulated immune response present in Kimura’s disease, induced by eosinophiliotrophic cytokines. However, the aetiology of IgG4‐RD is unclear and may have similar pathogenesis as Kimura’s disease: a combination of autoimmunity and allergic phenomenon with a subsequent T-cell response. IgG4-RD is a systemic, fibrosing condition that can affect any organ, especially the pancreas and salivary glands, causing tumefactive lesions. It is most frequent in individuals older than 50-years-old. Lymphadenopathy is also a common finding of this disease. Patients most commonly have multiorgan involvement, elevated serum IgG4, and may also exhibit eosinophilia and increased IgE levels. Pathological findings have morphological features that can be used for distinction, such as lymphoplasmacytic infiltration rich in IgG4 plasma cells, obliterative phlebitis and extensive fibrosis with a characteristic storiform pattern.^8,14^ Additional studies are needed to comprehend the relationship between both entities.

The treatment of this disease is controversial and can include surgery, radiotherapy and chemotherapy. Surgical excision alone is associated with high rates of relapse. A combination of surgery with low-dose radiotherapy may achieve the lowest local recurrence.^3^

Misdiagnosis of Kimura’s disease is not infrequent because it is often not suspected clinically. Although this rare disease is endemic mainly in parts of Asia, it should also be considered in the differential diagnosis of head and neck lesions in individuals of any descent, particularly in young males with peripheral eosinophilia and increased serum IgE.

## Learning points

- Kimura’s disease affects individuals of any descent. A detailed clinical history along with radiological and pathological correlation is critical for the correct diagnosis.
- The disease affects most commonly young males, and it is characterized by the presence of long-standing and painless subcutaneous masses in the head and neck region, swelling of major salivary glands, and lymphadenopathies.
- The salivary glands are frequently not affected in non-Asian descent patients.
- Peripheral eosinophilia, high serum IgE, and multiple lymphadenopathies are crucial findings that have consistently been associated with Kimura’s disease.
- Pathological assessment is the golden standard for the diagnosis and should be confirmed by surgical specimens.
- Surgical excision combined with low-dose radiation therapy may be the best treatment plan to reduce local recurrence.
